# P05.44. Evaluation of the Phlegm Syndrome Questionnaire: a new instruction to assess traditional Chinese medicine syndrome for angina

**DOI:** 10.1186/1472-6882-12-S1-P404

**Published:** 2012-06-12

**Authors:** Z Huiyong, Y Guanlin, Y Li, Y Changhe, Z Zhe, L Maoxin, P Yupeng

**Affiliations:** 1 Affiliated Hospital of Liaoning University of TCM, Shenyang, China; 2 Liaoning University of Traditional Chinese Medicine, Shenyang, China

## Purpose

We have established a draft version of a Phlegm Syndrome Questionnaire (PSQ) to assess the syndrome. The study is to assess the reliability and validity of the new questionnaire.

## Methods

The draft version was tested on a sample of 430 patients with angina, 178 (41.40%) were diagnosed with phlegm syndrome (PS). The Settle Angina Questionnaire (SAQ) was tested as well as an indicator of concurrent validity. A subset of patients (n=86) completed the questionnaire again 24 hours later to confirm test-retest reliability. The items were reduced by accessing item property. Reliablility was assessed via Cronbach's coefficient alpha. Exploratory factor analysis was performed to determine the number of domains. The discriminant validity was assessed by detecting differences between PS-identified patients and non-PS.

## Results

Test-retest correlation coefficient (CC) of the overall scale was 0.799, and each item ranged from 0.588 to 0.783, except for the “heavy head” item (0.528). The item was removed due to the lower contribution to the Cronbach’s alpha coefficient. The remained 8 items were as follow: bloating, tasteless, loss of appetite, heavy limbs, somnolence, sticky stool, sputum and sticking mouth. Three factors were extracted by exploratory factor analysis, and were stratified into 2 domains: spleen deficiency, heaviness and stickiness, which are the pathogenic characteristics of the phlegm. Cronbach’s alpha coefficient was 0.733, and for each domian are 0.648, 0.619. The CC between PSQ and SAQ was 0.414, and ranging from 0.128 to 0.366 for each items. There was significant difference between PS patients and non-PS in the overall scale and in each item (p<0.01), except for in “phlegm” (p=0.069) and ”loss of appetite” (0.153).

## Conclusion

The 8-item Phlegm Syndrome Questionnaire was developed utilizing sound psychometric properties, and can be used as an outcome in combination with SAQ for determining the treatment of angina in clinical practice.

